# Análise de Prevalência de Fibrilação Atrial e a Saúde Cardiovascular em Coorte Derivada do Projeto ELSA-Brasil

**DOI:** 10.36660/abc.20220676

**Published:** 2022-11-01

**Authors:** Henrique Tria Bianco

**Affiliations:** 1 Universidade Federal de São Paulo Escola Paulista de Medicina São Paulo SP Brasil Universidade Federal de São Paulo Escola Paulista de Medicina, São Paulo, SP – Brasil

**Keywords:** Fibrilação Atrial, Flutter Atrial, Fatores de Risco

Este interessante estudo^1^ de prevalência analisou a associação entre a fibrilação (FA) e o flutter atrial (FT), com o *status* da saúde cardiovascular, em coorte derivado do projeto ELSA-BRASIL, em desenho observacional e do tipo transversal. Foram incluídos nesta análise 13.141 participantes. Métricas de biomarcadores e dados epidemiológicos foram inseridos, com posterior análise de associação, em modelo ajustado e em regressão logística para as variáveis de interesse.

Os mecanismos de associação entre FA e o risco de acidente vascular cerebral (AVC) são bem conhecidos. A FA está associada à estase sanguínea anormal, que envolve hipocontratilidade atrial, remodelação estrutural atrial, ativação plaquetária e da cascata de coagulação, promovendo a formação de trombos e isquemia.^2,3^ Desta forma, a FA é preditor independente de doenças isquêmicas, notadamente o AVC. O risco estimado de FA durante a vida é entre 22% e 26%.^4,5^ Métricas para avaliar o impacto desta associação são constantemente publicadas em documentos, como as recomendações do Comitê de Metas e Métricas da Força-Tarefa de Planejamento Estratégico da *American Heart Association*, que desenvolveu estratégias de monitoramento contínuo e em longo prazo.^6^ Dentro de um conceito generalista, a saúde cardiovascular deve conter aspectos clínicos e comportamentais, como o estilo de vida adequado (não fumar, evitar a obesidade), concomitante a correção e adequação de biomarcadores metabólicos, como níveis de colesterol e triglicérides; glicemia; e controle adequado da pressão arterial. Desta forma, este comitê propôs um desafio para o alcance destas metas: “Até 2020, melhorar a saúde cardiovascular de todos os americanos em 20%, reduzindo as mortes por doenças cardiovasculares e AVC em 20%”. Desta forma, a identificação de indivíduos com risco para desenvolver FA é um imperativo clínico, pois a modificação de algumas variáveis pode reduzir a incidência desta afecção.^7,8^

Em estudo recentemente publicado, foi demonstrado que, em modelo utilizando “machine learning”, usando o eletrocardiograma (ECG) para estimar o risco de FA, foram robustos e validados em várias populações utilizando-se de rigorosas métricas epidemiológicas. A previsão de FA pode ser realizada pela avaliação dos fatores de risco clínicos ou análise de ECGs baseada em inteligência artificial. Entretanto, a combinação de ambos fornece maior precisão preditiva.^9^

No estudo intitulado: Saúde Cardiovascular e Fibrilação ou Flutter Atrial: Um Estudo Transversal do ELSA-Brasil,^1^ não foram observadas associações significativas entre os escores globais (saúde cardiovascular ideal) e o diagnóstico de FA, pelo menos parcialmente, em virtude de correlações antagônicas com a pressão arterial e com o colesterol total, dados estes extensivamente discutidos neste manuscrito. Este paradoxo entre o colesterol e a FA, foi consistente com dados previamente publicados. Em revisão sistemática, Guan et al. apontaram que níveis elevados de colesterol total (definidos em estudos com pontos de corte entre 220 e 260 mg/dL), estavam associados à FA, [HR 0,81 (IC 95%: 0,72-0,92)]. Na mesma revisão sistemática, análises usando LDL-C em vez de níveis de colesterol total, produziram resultados semelhantes.^10^ Entretanto, existem robustas evidências de que estatinas têm um benefício potencial na saúde incidência de FA ou FT.^11,12^ Desta maneira, métricas devem considerar um perfil não ideal de saúde cardiovascular se os pacientes estiveram sob medicação hipolipemiante, independentemente de seus níveis de colesterol total.

Com relação à hipertensão arterial, o presente manuscrito proveniente da coorte ELSA-BRASIL, analisou de forma separada as métricas dos escores globais de saúde. Os autores deste encontraram uma forte e inversa associação da pressão arterial com a presença de FA. Destacando desta forma, a relevância do controle e do tratamento adequado deste importante fator de risco, em consonância com dados da literatura.^13–15^

Os autores declaram algumas limitações que poderiam impactar nos resultados obtidos, como o pequeno número de participantes e sobretudo pela alta proporção de indivíduos com menos de 60 anos, havendo possível influência dos achados de “não associação” entre o diagnóstico FA e os escores de saúde global avaliados.
