# Supplementary material for: Análise de Prevalência de Fibrilação Atrial e a Saúde Cardiovascular em Coorte Derivada do Projeto ELSA-Brasil
Source: Arq Bras Cardiol. 2022 Nov 1;119(5):732–3. [Article in Portuguese] doi: 10.36660/abc.20220676 (PMC9750223; doi:10.36660/abc.20220676)
Supplement: Translation

# Translation: Analysis of Atrial Fibrillation Prevalence and Cardiovascular Health in Cohort Derived from the ELSA-Brasil Project

**Authors:** Henrique Tria Bianco

**Affiliations:** 1 São Paulo SP Brazil Universidade Federal de São Paulo Escola Paulista de Medicina, São Paulo, SP – Brazil

**Keywords:** Atrial Fibrillation, Atrial Flutter, Risk Factors

This interesting study^1^ of prevalence analyzed the association between atrial fibrillation (AF) and atrial flutter (AFL) with cardiovascular health status, in a cohort derived from the ELSA-BRASIL project, in an observational, cross-sectional design. A total of 13,141 participants were included in this analysis. Biomarker metrics and epidemiological data were entered with subsequent association analysis in an adjusted model and logistic regression for the variables of interest.

The mechanisms of association between AF and cerebrovascular accident (CVA) risk are well known. AF is associated with abnormal blood stasis that involves atrial hypocontractility, atrial structural remodeling, platelet activation and the coagulation cascade, promoting thrombus formation and ischemia.^2,3^ Thus, AF is an independent predictor of ischemic diseases, notably stroke. The estimated lifetime risk of AF is between 22% and 26%.^4,5^ Metrics to assess the impact of this association are constantly published in documents, such as the recommendations of the American Heart Association Strategic Planning Task Force Goals and Metrics Committee, which developed continuous and long-term monitoring strategies.^6^ Within a general concept, cardiovascular health must contain clinical and behavioral aspects, such as an adequate lifestyle (no smoking, avoiding obesity), concomitant with the correction and adequacy of metabolic biomarkers, such as cholesterol and triglyceride levels; blood glucose; and adequate blood pressure control. Thus, this committee proposed a challenge to reach these goals: “By 2020, improve the cardiovascular health of all Americans by 20%, reducing deaths from cardiovascular disease and stroke by 20%”. So, identifying individuals at risk for developing AF is a clinical imperative, as modifying some variables can reduce the incidence of this condition.^7,8^

In a recently published study, it was demonstrated that, in a model using “machine learning”, using the electrocardiogram (ECG) to estimate the risk of AF, they were robust and validated in several populations using rigorous epidemiological metrics. AF can be predicted by assessing clinical risk factors or analyzing ECGs based on artificial intelligence. However, the combination of both provides greater predictive accuracy.^9^

In the study entitled: Cardiovascular health and atrial fibrillation or flutter: A cross-sectional study from ELSA-Brasil,^1^ no significant associations were observed between global scores (ideal cardiovascular health) and the diagnosis of AF, at least partially due to antagonistic correlations with blood pressure and total cholesterol, data which are extensively discussed in this manuscript. This paradox between cholesterol and AF was consistent with previously published data. In a systematic review, Guan et al. pointed out that high levels of total cholesterol (defined in studies with cut-off points between 220 and 260 mg/dL) were associated with AF [HR 0.81 (95% CI: 0.72-0.92)]. In the same systematic review, analyses using LDL-C rather than total cholesterol levels produced similar results.^10^ However, there is robust evidence that statins have a potential health benefit in the incidence of AF or AFL.^11,12^ Therefore, metrics should consider a non-ideal cardiovascular health profile if patients were on lipid-lowering medication, regardless of their total cholesterol levels.

Regarding arterial hypertension, the present manuscript from the ELSA-BRASIL cohort analyzed the metrics of global health scores separately. The authors of this study found a strong and inverse association between blood pressure with the presence of AF. Thus, highlighting the relevance of control and adequate treatment of this important risk factor, in line with data in the literature.^13–15^

The authors state some limitations that could impact the results obtained, such as the small number of participants and, above all, the high proportion of individuals under 60 years, with a possible influence of the findings of “non-association” between the AF diagnosis and the assessed global health scores.
